# Minimally Invasive Suture Technique Pull-out to Repair the Acute Flexor Tendons in Zone II of the Hand

**DOI:** 10.1055/s-0044-1779332

**Published:** 2024-03-21

**Authors:** João Carlos Belloti, Luis Antonio Buendia, Marcel Jun Tamaoki, João Batista Gomes dos Santos, Flávio Falopa, Heitor José Rizardo Ulson

**Affiliations:** 1 Departamento de Ortopedia e Traumatologia, Universidade Federal de São Paulo, São Paulo, SP, Brasil; 2 Departamento de Ortopedia e Traumatologia, Hospital Municipal Carmino Caricchio, São Paulo, SP, Brasil; 3 Departamento de Ortopedia e Traumatologia, Hospital Samaritano de São Paulo, São Paulo, SP, Brasil

**Keywords:** tendon injuries, rupture, suture

## Abstract

**Objective:** To evaluate the safety and effectiveness of a modified pull-out suture technique in patients undergoing primary repair surgery for injuries to the flexor tendons of the fingers with Total Active Motion (TAM) as the primary outcome.

**Method:** A total of 29 patients (38 fingers) were chosen from both sexes, aged between 18 and 65 years with clean acute tendon laceration occurring within 15 days, in the Verdan's zone II of flexor tendon in the hand, when only the deep flexor tendon was sutured, either associated or not with digital nerve injury. The patients were operated on using the proposed technique and evaluated at 3, 9 and 24-weeks PO. The primary outcome was the assessment of Total Active Movement (TAM) and 3 classifications were employed: Strickland, IFSSH and Buck-Gramcko.

**Results:** We observed a total active motion (TAM) of 209.3 °at the end of 24 weeks; 83.0% of Good and Excellent results by the Modified Strickland Classification, 93% of Excellent results by the IFSSH Classification, and 97% of Good and Excellent results using the Buck-Gramcko Classification. There were no cases of rupture, but tendon adhesion was observed in 3 fingers.

**Conclusion:** The present suture technique proved to be safe and effective with a low rate of complications, obtaining an excellent functional result in terms of total active mobility, according to the evaluations and classifications used.

## Introduction

Injuries to the flexor tendons in zone II most often affect economically active individuals with a predominance of male patients (84.1%). 1 About 13 to 19% of these lesions are susceptible to complications, with the most frequent being adhesions (9–13%) and re-ruptures (4–6%). 2 Prevention of adhesion formation in the osteofibrous tunnel is a challenge for surgeons and can cause reduced joint mobility of the affected finger and consequent joint contracture and grip deficit. 2

The best results for the surgical repair of acute injuries to the flexor tendons in zone II of the hand is mainly dependent on early treatment, 3 suture techniques that avoid gap formation providing sufficient strength to allow both active and passive mobility (four-thread flexor tendon repair has an estimated tensile strength of 50-110 N), 4 and that do not hamper the tendon slide through the osteofibrous tunnel. 5

Several suturing techniques as well as rehabilitation protocols have been described in literature. Although two-strand suture methods (Kessler and modified Kessler) still acceptance, newer multistrand suture technique are being used because they increased resistance to repair site gapping, 4 5 6 7 8 there is no conclusive evidence regarding the most effective ones. 9

We have developed a type of suture for the treatment of acute injuries of the flexor tendons in zone 2 of the hand that carries a pull-out suture applied through a mini-incision, which we call the MOP- Mini Open pull-out technique.

This technique proposes a suture with sufficient resistance to allow early active mobility, with a self-applied rehabilitation program carried out mostly at home under the supervision of a hand therapist during periodic appointments.

Our hypothesis was that the MOP technique can reduce the possible complications, the days off work and thecosts. In this study, we describe the MOP technique and the results of the treatment of 38 fingers in 29 patients.

## Method

### Research Design

This prospective study was carried out between April 2020 and May 2022, when the development of the surgical technique and the operations were carried out in their entirety by the two senior authors as approved by the ethics committee of the institutions participating, the patients signed an informed consent form. The surgical technique was initially developed in the anatomy laboratory, using the concepts previously described in the literature regarding both the pull-out technique and suture resistance for flexor tendons in zone II of the hand. 10 11

For the sample size calculation, we considered the primary outcome of the study as the TAM and its recovery during the third, ninth, and twenty-fourth weeks, with 95% as the confidence interval for the statistical power.

We included 29 adult patients (38 fingers) aged between 18 and 65 years of both sexes with clean acute tendon laceration occurring within 15 days, in Verdan's zone II of the deep flexor tendon in the hand, either associated or not with the digital nerve injury. Patients with untidy wounds, crush injuries, or injuries associated with fractures were not included in this study. To analyze the results and normality of the distribution of the population sample, we used the Shapiro-Wilk test. The patients' demographic profile was evaluated according to descriptive statistics and frequency, considering sex, age, affected side, hand dominance, associated injuries, accident location, and causing instrument.

To assess the primary outcome, we adopted the goniometric measurement of the metacarpophalangeal (MP), proximal interphalangeal (PIP) and distal interphalangeal (DIP) joints in their active and passive mobility in flexion and extension. These data available, we used four functional tests to evaluate the results, namely: TAM, Modified Strickland classifications, 12 IFSSH Classification (International Federation of Societies of Surgery of the Hand) 3 and the Buck-Gramcko classification. 13

The primary TAM outcome measures were collected at three time points after surgery (third, ninth, and 24th postoperative weeks), and One-Way Repeated Measures ANOVA was applied to evaluate the mean finger difference in mobility postoperatively

### Description of Surgical Technique

**Fig. 1:**
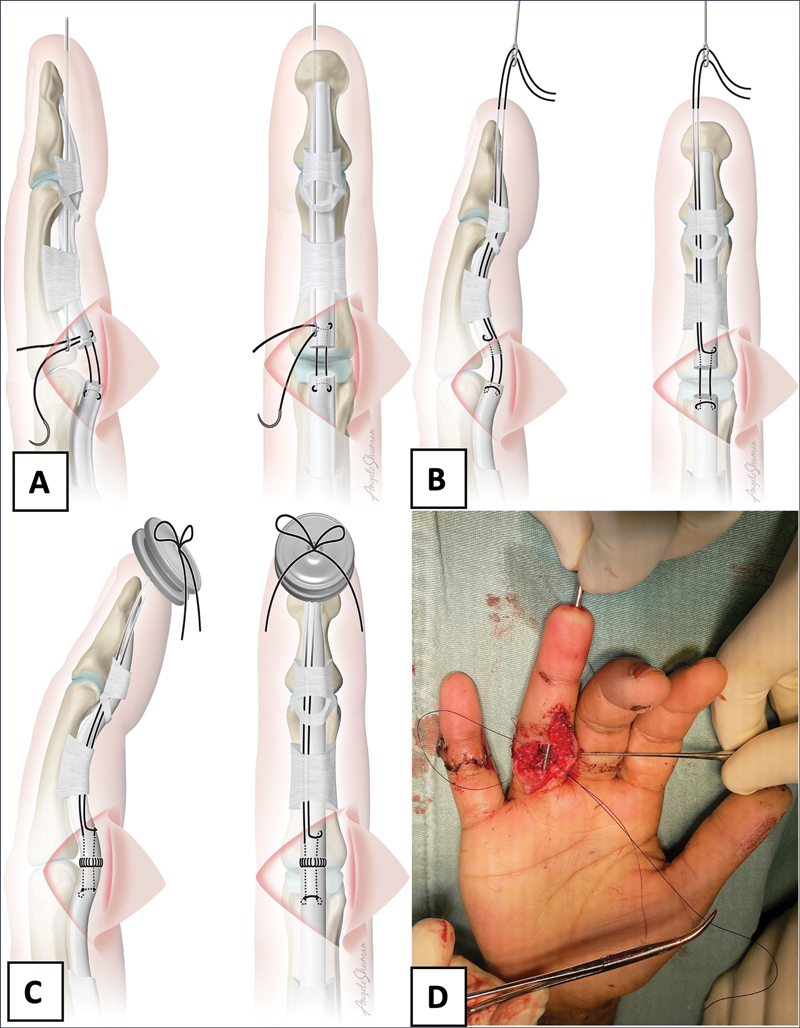
A) Front and lateral view with the thread passer introduced inside the osteofibrous tunnel with the suture thread passing through the holes of the passer. B) The lateral and front view when pulling the thread passer that is inside the osteofibrous tunnel to the digital tip carrying the suture thread performed after making the Kessler tie of flexor digitorum profundus (FDP). C, D) Making the suture knot over the silicone shield.

**Fig. 2:**
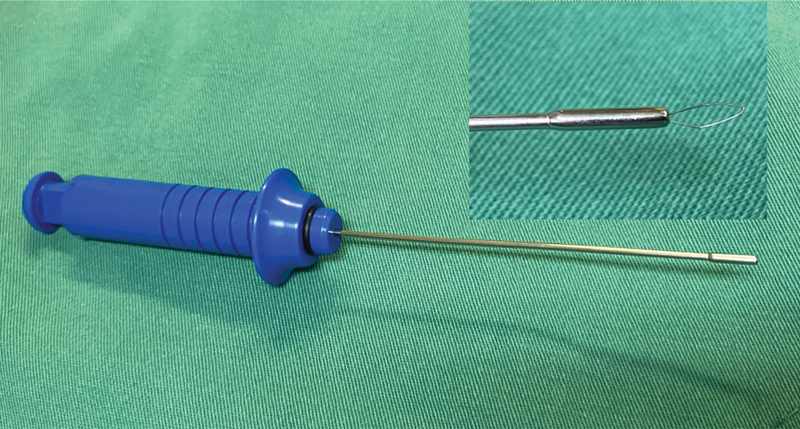
A) Final minimally invasive appearance after skin suturing. B) The surgical drapes are lowered so that the patient may observe the passive movement of the injured finger by the surgeon, and we stimulate active movement by the patient.

The surgical procedure was performed using the WALANT anesthesia technique. 14 The access approach was used with minimum magnification as necessary for the exploration of the wound and visualization of the tendinous stumps and digital nerves. Only the deep flexor tendon was sutured. Used a 3-0 mononylon thread in a modified Kessler-type suture 15 16 applied to the proximal stump and distal stump, leaving the suture thread ends free to be guided for the pullout fixation on the digital pulp tip ( Fig. 1A ). We made a small incision on the digital pump enough to introduce a 1.4 mm diameter special suture passer (developed by authors) ( Fig. 2 ). The threads were passed through the osteofibrous tunnel to the digital pulp whit the special suture passer ( Fig. 1B-D ). Suture fixation was done on a silicone button to protect the soft parts of the digital pulp and under adequate tension in flexion ( Fig. 1C ). The procedure was completed using an epitendinous suture with 5-0 mononylon thread ( Fig. 1C ). The result of this technique is that the 3.0 nylon thread (Kessler suture) slides outside and parallel to the tendon but within the osteofibrous tunnel so that the repair zone is free from direct traction forces that are shifted to the pull-out suture in the digital pulp as the finger is either flexed or extended. After finishing the suture, the patient is asked to actively move the finger to assess the free sliding of the tendon in the osteofibrous tunnel if there were no gap/s, and if the suture of the digital nerve, when repaired, is free of tension. 17 It is also important to assess if the tension of the MOP suture is adequate, and if the patient performs the full range of active flexion-extension without difficulties ( Fig. 2A-B ). During the immediate postoperative period, the patient received guidance from the hand therapist on how to carry out home-based rehabilitation and a protocol for the same with guidelines ( Table 1 ). A simple wrapping is applied to protects the wound without using any kind of mobilization. Each patient's return was scheduled for one week after the operation and the Silicone button is removed at 8 weeks along with the pull out.

**Table 1:** Protocol for home rehabilitation

Guidelines After Hand Tendon Repair Surgery 1. To keep the operated upper limb elevated with fingers pointing upwards and to avoid keeping the hand below the chest 2. To perform the following finger exercises, especially with the operated finger, three times a day (morning, afternoon, and evening): 2a- Completely open and close all fingers with the help of the non-operated hand, a total of 15 times 2b. Open and close all fingers of the operated hand without the help of the other hand, a total of 15 times 3. To avoid carrying weight (bags or heavy objects) and not perform exercises to squeeze objects (balls, springs, etc.) 4. Not to remove or pull on the button that is on the fingertip.

## Results

The results of the 38 fingers in 29 adult patients were as follows: 11 women (37.94%) and 18 men (62.06%), with a minimum age of 18 years and a maximum age of 65 years, with a mean age of 38.5 years. Three patients had two fingers sutured and three patients had injuries in three fingers of the same hand; no patient had flexor tendon injuries in both hands. A total of 27 fingers were affected on the right side (71.85%) and 11 on the left side (28.95%). Of these, 28 of the injured fingers were of the dominant hand (73.68%), and the other 10 fingers were on the non-dominant hand. The index finger was the most affected, with 11 tendon injuries (28.94%), followed by the ring, middle and little fingers with 8 injuries each (21.05%). Finally, the thumb was affected in 3 patients (7.89%) ( Table 2 ).

**Table 2:** Distribution of Qualitative Variables

		N	%
Affected finger	Thumb	3	7,89
	Index	11	28,94
	Middle	8	21,05
	Ring	8	21,05
	Small	8	21,05
Gender	Female	11	39,47
	Male	18	60,52
Affected side	Right	27	71,05
	Left	11	28,95
Digital nerve injury	Radial	5	13,51
	Ulnar	6	16,21
Dominant hand	No	9	31,03
	Yes	20	68,96

### Total Active Movement

**Fig. 3:**
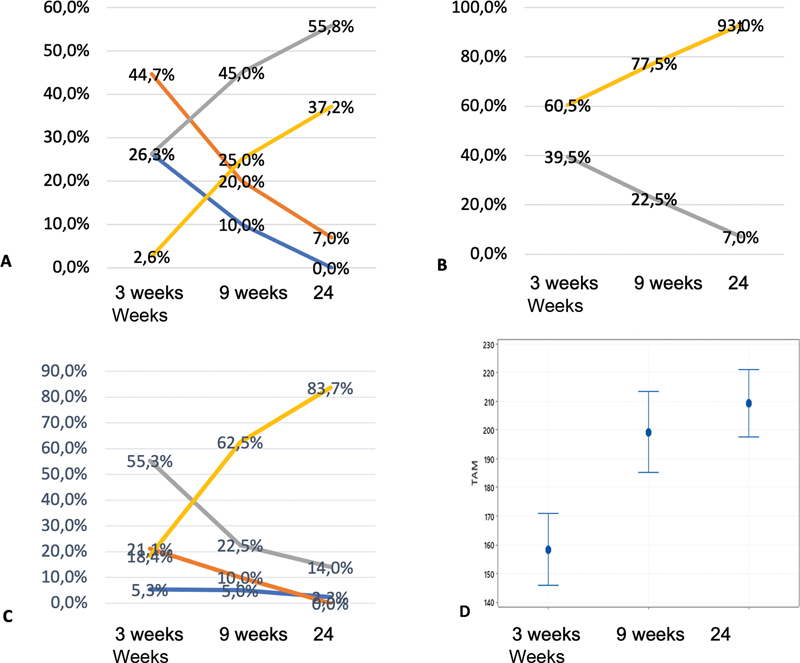
A) Results of the Modified Strickland classification. B) Results of the IFSSH. C) Results of the Buck-Gramcko classification. D) The results for Total Active Mobility (TAM).

The mean TAM was 158° in the 3rd week, 199° in the 9th week and 209° in the 24th week postoperatively. The greatest gain in TAM (40.7°) occurred between the third and ninth postoperative weeks ( Table 3 and Fig. 3D ).

**Table 3:** List of times when assessments were carried out in Total Active Mobility (TAM)

Evolution		Mean	Median	Standard Deviation	Q1	Q3	N	IC	P-valor
TAM	3 Sem	158,4	155	36,0	140	180	35	11,9	<0,001
	9 Sem	199,1	195	41,0	180	220	35	13,6	
	24 Sem	209,3	210	34,1	193	235	35	11,3	

### Strickland Modified Classification

In the final evaluation using the modified Strickland classification at 24 weeks, we observed regular (7.0%), good (55.8%), and excellent (37.2%) results ( Table 4 and Fig. 3A ).

**Table 4:** List of evaluation moments in the 3 classifications

		3 weeks		9 weeks		24 weeks		Total		*p* value
		N	%	N	%	N	%	N	%	
IFSSH	Good	15	39,5%	9	22,5%	3	7,0%	27	22,3%	0,002
	Excellent	23	60,5%	31	77,5%	35	93,0%	94	77,7%	
Modified Strickland	Poor	10	26,3%	4	10,0%	0	0,0%	14	11,6%	<0,001
	Regular	17	44,7%	8	20,0%	3	7,0%	28	23,1%	
	Good	10	26,3%	18	45,0%	21	55,8%	52	43,0%	
	Excellent	1	2,6%	10	25,0%	14	37,2%	27	22,3%	
Buck-Gramcko	Poor	2	5,3%	2	5,0%	1	2,3%	5	4,1%	<0,001
	Regular	8 o	21,1%	4	10,0%	0	0,0%	12	9,9%	
	Good	21	55,3%	9	22,5%	5	14,0%	36	29,8%	
	Excellent	7	18,4%	25	62,5%	32	83,7%	68	56,2%	

### International Federation of Societies for Surgery of the Hand

The result of the IFSSH classification, we observed Good results (7.0%) in the 24th week. We observed Excellent results at 60.5% in the 3rd week which rose to 77.5% in the 9th week and ended at 93.0% in the 24th week ( Table 4 and Fig. 3B ).

### Buck-Gramcko

Evaluation with the Buck-Gramcko classification yielded precarious results with 2.3% in the 24th week; regular results at 0% in the 24th week, Good results at 14.0% in the 24th week, and Excellent results at 83.7% in the 24th week. There was a statistically significant difference between the time points ( Table 4 and Fig. 3C ).

### Modified Dellon-Sensitive Recovery

Of the patients who suffered damage to the associated digital nerve (11 patients, 29,72% of the sample), in the final evaluation (24th week PO), we had one patient that showed recovery in deep pain sensitivity (S1), corresponding to 9.09%; in three patients, there was an improvement in tactile sensitivity (S2) of 27.27%, six patients (54.54%) showed an S3 recovery (discrimination between two points 7 to 15 mm) and 1 patient (9.09%) showed S4 recovery (discrimination between two points from 2 to 6 mm).

## Complication

In 3 fingers we had as a complication the formation of tendinous adherence (7.89%), observed in two different patients: one patient (male 59 years old) the affected was the index finger, which presented a deficit of active extension of the MF, PIP and DIP of 40°, functional assessment at the 9th week; this patient underwent a new procedure: tenolysis after the 24th week, obtaining a good result after the surgery. The other pacient (38-year-old female), presented active extension deficit of 60° from the MF, PIP and DIP joints in the middle finger and an active extension deficit of 70° in the ring finger, observed in the evaluation at the 9th week, which did not want to undergo a new procedure treatment, she refused further surgery for being pregnant.

We had no case of re-rupture.

## Discussion

Although there are several suturing techniques for the repair of zone II flexor tendons of the hand, there is no consensus on the most effective.

Strickland proposes as an ideal method of suture an easy-to- perform with fixed knots, regular transverse facing, minimal to no gaps at the repair site, avoiding injury to the tendon vascularization, and having sufficient strength for the first active movements. 12 18 In an attempt to increase the resistance of the repair, several techniques (four strand, six strand) were used 19 although with the disadvantage of a possible increase in the volume and ischemia of the tendon, which can contribute to a lower slippage 20 and made tendon healing difficult.

The pull-out technique, proposed by Brunelli 10 21 22 consists of a central suture, starting at the lesion site, sliding inside the tendon substance, covering approximately 1.5 cm proximal to the lesion, and emerging distally in the digital pulp, transmitting the tension from the suture knot to the fingertip (pull-out) allowing immediate mobilization. In the modification we performed, the MOP technique used in this study; the central suture is performed observing the same principle as the technique proposed by Brunelli except our suture thread arms run through the osteo-fibrous tunnel parallel to the tendon but not inside the tendon proper, and having the suture knot tied on a silicone button placed at the digital pulp, then a circumferential epitendinous suture is applied to the tendon ends. Strickland recommends that both tendons in zone 2 injuries must be repaired, 12 however, some cadaveric studies have shown that repairing the superficialis increases slip work in bottom repair alone, and repairing just one slip of the superficialis may be beneficial in reducing the flexion work. In addition, Tang has shown that there was no difference in total active movement in the repair of both the deep and the superficial in zone 2 versus deep repair alone, 7 and that in the group that had both repaired tendons ended up having more reoperations because of adhesions. 23 Another differential of the MOP technique is the use of the wire guide that is introduced through the incision in the finger pulp, avoiding extending skin incisions, as well as venting the Pulleys A2 and A4, avoiding damage soft tissue and reducing the possible “bowstring” effect. 23 The use of local anesthesia using the WALANT technique, with the patient awake, allows us to observe the active movement of the finger to check for possible failure of tendon slippage or gap formation, and when necessary, make adjustments to the suture tension before skin closure. 24

The use of orthosis or some type of immobilization is a consensus in almost all studies on tenorrhaphy rehabilitation techniques in zone II of the hand. 9

Recommended types of rehabilitation are generally based on the nature of the injury, stage of rehabilitation (immediately versus immobilization regimen), the strength of the repair (number of sutures in the repair), associated injuries, or the ability to comply with rehabilitation. 25

One of the proposals of the MOP technique is to start with both active and passive movements of the injured finger(s), through guidance from the surgical team during the intraoperative period when the patient can visualize and perform flexion and extension movements of the finger(s) repaired, which will later be performed by the patient himself through a home rehabilitation protocol ( Table 1 ), without the need to use orthoses, which greatly facilitated the rehabilitation of our patient's environment.

In this study, we prospectively evaluated the MOP technique, performed by two surgeons in two centers. There is a need to evaluate its effectiveness through prospective randomized studies comparing its results with other suturing techniques long established in the literature, in a multicentric manner and carrying a greater number of surgeons, in order to verify its reproducibility and external validity. Our results demonstrate that the MOP technique can be another option within the therapeutic arsenal for these lesions. The MOP suture technique for repairing the flexor tendons in zone II of the hand has been shown to be safe and effective with a low complication rate. 26
