# Supplementary material for: Minimally Invasive Suture Technique Pull-out to Repair the Acute Flexor Tendons in Zone II of the Hand
Source: Rev Bras Ortop (Sao Paulo). 2024 Mar 21;59(1):e60–7. doi: 10.1055/s-0044-1779332 (PMC10957262; doi:10.1055/s-0044-1779332)
Supplement: Related texts

# Research article: Técnica de sutura minimamente invasiva pull-out para reparar lesões agudas dos tendões flexores da zona II da mão

**Keywords:** lesões de tendões, ruptura, sutura

## Abstract

**Objetivo:** Avaliar a segurança e a efetividade da técnica de sutura pull-out modificada nos pacientes submetidos à cirurgia de reparo primário das lesões dos tendões flexores nos dedos, tendo como desfecho primário o movimento ativo total (TAM).

**Método:** Foram escolhidos 29 pacientes (38 dedos), de ambos os sexos, com idade entre 18 e 65 anos, com laceração tendinosa aguda e limpa, ocorrida em até 15 dias, na zona II de Verdan do tendão flexor da mão, quando apenas o tendão flexor profundo foi suturado, associada ou não à lesão do nervo digital. Os pacientes foram operados pela técnica proposta e avaliados em 3, 9 e 24 semanas PO. O desfecho primário foi a avaliação do movimento ativo total (TAM) e foram empregadas 3 classificações: Strickland, IFSSH e Buck-Gramcko.

**Resultados:** Observamos um movimento ativo total (TAM) de 209,3° ao final de 24 semanas; 83,0% de resultados Bons e Excelentes pela Classificação de Strickland Modificada, 93% de resultados Excelentes pela Classificação IFSSH e 97% de resultados Bons e Excelentes pela Classificação Buck-Gramcko. Não houve casos de ruptura, mas foi observada aderência tendínea em 3 dedos.

**Conclusão:** A presente técnica de sutura mostrou-se segura e eficaz com baixo índice de complicações, obtendo excelente resultado funcional em termos de mobilidade ativa total, de acordo com as avaliações e classificações utilizadas.

## Introdução

A presente técnica de sutura mostrou-se segura e eficaz com baixo índice de complicações, obtendo excelente resultado funcional em termos de mobilidade ativa total, de acordo com as avaliações e classificações utilizadas. 2 A prevenção da formação de aderências no túnel osteofibroso é um desafio para os cirurgiões e pode causar redução da mobilidade articular do dedo afetado e consequente contratura articular e déficit de preensão. 2

Os melhores resultados para a reparação cirúrgica das lesões agudas dos tendões flexores da zona II da mão dependem principalmente do tratamento precoce, 3 técnicas de sutura que evitem a formação de lacunas e forneçam força suficiente para permitir a mobilidade ativa e passiva (reparo do tendão flexor de quatro fios tem uma resistência à tração estimada de 50-110 N), 4 e que não dificultam o deslizamento do tendão através do túnel osteofibroso. 5

Diversas técnicas de sutura, bem como protocolos de reabilitação foram descritos na literatura. Embora os métodos de sutura de dois fios (Kessler e Kessler modificado) ainda sejam aceitos, novas técnicas de sutura de múltiplos fios estão sendo usadas porque aumentam a resistência ao reparo de lacunas no local, 4 5 6 7 8 não há evidências conclusivas sobre quais são as mais eficazes. 9

Desenvolvemos um tipo de sutura para tratamento de lesões agudas dos tendões flexores da zona II da mão que realiza uma sutura pull-out aplicada através de uma mini-incisão, que chamamos de técnica MOP - mini open pull-out.

Esta técnica propõe uma sutura com resistência suficiente para permitir a mobilidade ativa precoce, com um programa de reabilitação autoaplicado realizado maioritariamente em casa sob supervisão de um terapeuta da mão em consultas periódicas.

Nossa hipótese foi que a técnica MOP pode reduzir as possíveis complicações, as faltas ao trabalho e os custos. Neste estudo descrevemos a técnica MOP e os resultados do tratamento de 38 dedos em 29 pacientes.

## Método

## Planejamento da pesquisa

Este estudo prospectivo foi realizado entre abril de 2020 e maio de 2022, quando o desenvolvimento da técnica cirúrgica e as operações foram realizadas na íntegra pelos dois autores seniores conforme aprovação do comitê de ética das instituições participantes, os pacientes assinaram um termo de consentimento informado. A técnica cirúrgica foi desenvolvida inicialmente no laboratório de anatomia, utilizando os conceitos já descritos na literatura tanto sobre a técnica de pull-out quanto sobre resistência de sutura para tendões flexores da zona II da mão. 10 11

Para o cálculo do tamanho da amostra, considerou-se como desfecho primário do estudo o TAM e sua recuperação durante a terceira, nona e vigésima quarta semanas, tendo 95% como intervalo de confiança para o poder estatístico.

Foram incluídos 29 pacientes adultos (38 dedos), com idade entre 18 e 65 anos, de ambos os sexos, com laceração tendinosa aguda e limpa, ocorrida há 15 dias, do tendão flexor profundo na zona II de Verdan da mão, associada ou não à lesão do nervo digital. Pacientes com feridas não tratadas, lesões por esmagamento ou lesões associadas a fraturas não foram incluídos neste estudo. Para analisar os resultados e a normalidade da distribuição da amostra populacional, utilizou-se o teste de Shapiro-Wilk. O perfil demográfico dos pacientes foi avaliado segundo estatística descritiva e frequência, considerando sexo, idade, lado acometido, dominância de mão, lesões associadas, local do acidente e instrumento causador.

Para avaliar o desfecho primário, adotamos a medida goniométrica das articulações metacarpofalângica (MCF), interfalângica proximal (IFP) e interfalângica distal (IFD) em sua mobilidade ativa e passiva em flexão e extensão. Com esses dados disponíveis, utilizamos quatro testes funcionais para avaliar os resultados, a saber: TAM, classificações de Strickland modificadas, 12 classificação IFSSH ( *International Federation of Societies of Surgery of the Hand* ) e a classificação de Buck-Gramcko. 13

As medidas de resultados primários do TAM foram coletadas em três momentos após a cirurgia (terceira, nona e 24ª semanas de pós-operatório), e a ANOVA de medidas repetidas unidirecionais foi aplicada para avaliar a diferença média dos dedos na mobilidade no pós-operatório.

## Descrição da Técnica Cirúrgica

Foi utilizada uma abordagem de acesso com a ampliação mínima necessária para a exploração da ferida e visualização das extremidades tendinosas e nervos digitais. Apenas o tendão flexor profundo foi suturado. Foi usada uma linha mononylon 3-0 em uma sutura tipo Kessler modificada 15 16 aplicada na extremidade proximal e distal, deixando as pontas da linha de sutura livres para serem guiadas para a fixação por tração na ponta da polpa digital ( Fig. 1A ). Realizamos uma pequena incisão na polpa digital suficiente para introduzir um passador de sutura especial de 1,4 mm de diâmetro (desenvolvido pelos autores) ( Fig. 2 ). Os fios foram passados através do túnel osteofibroso até a polpa digital com o passador de sutura especial ( Fig. 1B-D ). A fixação da sutura foi realizada em um botão de silicone para proteger as partes moles da polpa digital e sob tensão adequada em flexão ( Fig. 1C ). O procedimento foi concluído com uma sutura epitendinosa usando linha mononylon 5-0 ( Fig. 1C ). O resultado dessa técnica é que a linha de nylon 3.0 (sutura Kessler) desliza do lado de fora e paralela ao tendão, mas dentro do túnel osteofibroso, de modo que a zona de reparo fica livre de forças de tração direta que são transferidas para a sutura de tração na polpa digital à medida que o dedo é flexionado ou estendido. Após o término da sutura, pede-se ao paciente que mova ativamente o dedo para avaliar o deslizamento livre do tendão no túnel osteofibroso, se não houver aberturas, e se a sutura do nervo digital, quando reparada, estiver livre de tensão. 17 Também é importante avaliar se a tensão da sutura MOP é adequada e se o paciente realiza toda a amplitude de flexão-extensão ativa sem dificuldades ( Fig. 2 A-B ). Durante o período pós-operatório imediato, o paciente recebeu orientações do terapeuta da mão sobre como realizar a reabilitação em casa, juntamente com um protocolo contendo diretrizes para o mesmo ( Tabela 1 ). Um simples envoltório é aplicado para proteger a ferida sem utilizar qualquer tipo de mobilização. O retorno de cada paciente foi agendado para uma semana após a operação e o botão de silicone é retirado com 8 semanas junto com o pull out.

**Fig. 1:** A) Vista frontal e lateral com o passador de fio introduzido dentro do túnel osteofibroso com o fio de sutura passando pelos orifícios do passador. B) Vista lateral e frontal ao puxar o fio passador que fica dentro do túnel osteofibroso até a ponta digital portando o fio de sutura realizado após confecção do laço Kessler do flexor profundo dos dedos (FDP). C, D) Fazendo o nó de sutura sobre a proteção de silicone.

**Fig. 2:** A) Aspecto final minimamente invasivo após sutura da pele. B) Os campos cirúrgicos são abaixados para que o paciente observe a movimentação passiva do dedo lesionado pelo cirurgião, e estimulamos a movimentação ativa do paciente.

**Tabela 1:** Protocolo para reabilitação domiciliar

Orientações Pós Cirurgia de Reparo de Tendão na Mão 1. Manter o membro superior operado elevado, com os dedos apontando para cima, e evitar mantê-lo abaixo do peito. 2. Realizar os seguintes exercícios com os dedos, especialmente com o dedo operado, três vezes ao dia (manhã, tarde e noite): 2a. Abrir e fechar completamente todos os dedos com a ajuda da mão não operada, um total de 15 vezes. 2b. Abrir e fechar todos os dedos da mão operada sem a ajuda da outra mão, um total de 15 vezes. 3. Evitar carregar peso (bolsas ou objetos pesados) e não realizar exercícios de apertar objetos (bolas, molas, etc.). 4. Não remover ou puxar o botão que está na ponta do dedo.

## Resultados

Os resultados dos 38 dedos em 29 pacientes adultos foram os seguintes: 11 mulheres (37,94%) e 18 homens (62,06%), com idade mínima de 18 anos e máxima de 65 anos, com média de idade de 38,5 anos. Três pacientes tiveram dois dedos suturados e três pacientes tiveram lesões em três dedos da mesma mão; nenhum paciente apresentou lesão nos tendões flexores de ambas as mãos. Foram acometidos 27 dedos no lado direito (71,85%) e 11 no lado esquerdo (28,95%). Destes, 28 dedos lesionados eram da mão dominante (73,68%) e os outros 10 dedos eram da mão não dominante. O dedo indicador foi o mais acometido, com 11 lesões tendíneas (28,94%), seguido dos dedos anular, médio e mínimo com 8 lesões cada (21,05%). Por fim, o polegar foi acometido em 3 pacientes (7,89%) ( Tabela 2 ).

**Tabela 2:** Distribuição de Variáveis Qualitativas

		N	%
Dedo afetado	Polegar	3	7,89
	Indicador	11	28,94
	Médio	8	21,05
	Anelar	8	21,05
	Mínimo	8	21,05
Gênero	Feminino	11	39,47
	Masculino	18	60,52
Lado afetado	Direito	27	71,05
	Esquerdo	11	28,95
Lesão do nervo digital	Radial	5	13,51
	Ulnar	6	16,21
Mão dominante	Não	9	31,03
	Sim	20	68,96

## Movimento ativo total

A média do TAM foi de 158° na 3ª semana, 199° na 9ª semana e 209° na 24ª semana de pós-operatório. O maior ganho no TAM (40,7°) ocorreu entre a terceira e a nona semana de pós-operatório ( Tabela 3 e Fig. 3D ).

**Tabela 3:** Lista de momentos em que foram realizadas avaliações do Movimento Ativo Total (TAM)

Evolução		Média	Mediana	Desvio padrão	Q1	Q3	N	CI	P-valor
TAM	3 Sem	158,4	155	36,0	140	180	35	11,9	<0,001
	9 Sem	199,1	195	41,0	180	220	35	13,6	
	24 Sem	209,3	210	34,1	193	235	35	11,3	

**Fig. 3:** A) Resultados da classificação de Strickland Modificada. B) Resultados do IFSSH. C) Resultados da classificação de Buck-Gramcko. D) Resultados para Movimento Ativo Total (TAM).

## Classificação modificada de Strickland

Na avaliação final pela classificação de Strickland modificada às 24 semanas, observamos resultados regulares (7,0%), bons (55,8%) e excelentes (37,2%) ( Tabela 4 e Fig. 3A ).

**Tabela 4:** Lista dos momentos de avaliação nas 3 classificações

		3 semanas		9 semanas		24 semanas		Total		Valor de *p*
		N	%	N	%	N	%	N	%	
IFSSH	Bom	15	39,5%	9	22,5%	3	7,0%	27	22,3%	0,002
	Excelente	23	60,5%	31	77,5%	35	93,0%	94	77,7%	
Strickland modificada	Pobre	10	26,3%	4	10,0%	0	0,0%	14	11,6%	<0,001
	Regular	17	44,7%	8	20,0%	3	7,0%	28	23,1%	
	Bom	10	26,3%	18	45,0%	21	55,8%	52	43,0%	
	Excelente	1	2,6%	10	25,0%	14	37,2%	27	22,3%	
Buck-Gramcko	Pobre	2	5,3%	2	5,0%	1	2,3%	5	4,1%	<0,001
	Regular	8 o	21,1%	4	10,0%	0	0,0%	12	9,9%	
	Bom	21	55,3%	9	22,5%	5	14,0%	36	29,8%	
	Excelente	7	18,4%	25	62,5%	32	83,7%	68	56,2%	

## IFSSH (International Federation of Societies for Surgery of the Hand)

No resultado da classificação IFSSH, observamos bons resultados (7,0%) na 24ª semana. Observamos excelentes resultados de 60,5% na 3ª semana que subiu para 77,5% na 9ª semana e terminou em 93,0% na 24ª semana ( Tabela 4 e Fig. 3B ).

## Buck-Gramcko

A avaliação com a classificação de Buck-Gramcko apresentou resultados precários com 2,3% na 24ª semana; resultados regulares de 0% na 24ª semana, bons resultados de 14,0% na 24ª semana e resultados excelentes de 83,7% na 24ª semana. Houve diferença estatisticamente significativa entre os momentos ( Tabela 4 e Fig. 3C ).

## Recuperação Sensível de Dellon Modificada

Dos pacientes que sofreram lesão do nervo digital associado (11 pacientes, 29,72% da amostra), na avaliação final (24ª semana PO), tivemos um paciente que apresentou recuperação da sensibilidade dolorosa profunda (S1), correspondente para 9,09%; em três pacientes houve melhora da sensibilidade tátil (S2) de 27,27%, seis pacientes (54,54%) apresentaram recuperação S3 (discriminação entre dois pontos 7 a 15 mm) e 1 paciente (9,09%) apresentou recuperação S4 (discriminação entre dois pontos de 2 a 6 mm).

## Complicação

Em 3 dedos tivemos como complicação a formação de aderência tendinosa (7,89%), observada em dois pacientes diferentes: em um paciente (homem, 59 anos) o acometido foi o dedo indicador, que apresentava déficit de extensão ativa do MCF, IFP e IFD de 40°, avaliação funcional na 9ª semana; esse paciente foi submetido a novo procedimento: tenólise após a 24ª semana, obtendo bom resultado após a cirurgia. A outra paciente (feminino, 38 anos) apresentava déficit de extensão ativa de 60° das articulações MCF, IFP e IFD no dedo médio e déficit de extensão ativa de 70° no dedo anular, observado na avaliação de 9ª semana, que não quis fazer novo procedimento de tratamento, recusou nova cirurgia por estar grávida.

Não tivemos nenhum caso de re-ruptura.

## Discussão

Embora existam diversas técnicas de sutura para reparo dos tendões flexores da zona II da mão, não há consenso sobre qual é a mais eficaz.

Strickland propõe como método ideal de sutura um método de fácil execução, com nós fixos, face transversal regular, mínimo ou nenhum espaço no local do reparo, evitando lesão da vascularização do tendão e com força suficiente para os primeiros movimentos ativos. 12 18 Na tentativa de aumentar a resistência do reparo, várias técnicas (quatro fios, seis fios) foram utilizadas, 19 embora com a desvantagem de um possível aumento no volume e isquemia do tendão, o que pode contribuir para um menor deslizamento 20 e dificultar a cicatrização do tendão.

A técnica pull-out, proposta por Brunelli, 10 21 22 consiste em uma sutura central, iniciando no local da lesão, deslizando dentro da substância do tendão, cobrindo aproximadamente 1,5 cm proximal à lesão e emergindo distalmente na polpa digital, transmitindo a tensão do nó da sutura até a ponta dos dedos (tração), permitindo a mobilização imediata. Na modificação realizada neste estudo, a técnica MOP utilizada; a sutura central é realizada observando o mesmo princípio da técnica proposta por Brunelli, exceto que os braços da linha de sutura passam pelo túnel osteofibroso paralelamente ao tendão, mas não dentro do tendão propriamente dito, e o nó da sutura é amarrado em um botão de silicone colocado na polpa digital, em seguida, uma sutura epitendinosa circunferencial é aplicada nas extremidades do tendão. Strickland recomenda que ambos os tendões em lesões na zona II sejam reparados, 12 no entanto, alguns estudos em cadáveres mostraram que reparar o tendão superficial aumenta o trabalho de deslizamento na reparação inferior isolada, e reparar apenas um deslizamento do tendão superficial pode ser benéfico na redução do trabalho de flexão. Além disso, Tang mostrou que não houve diferença no movimento ativo total no reparo tanto do profundo quanto do superficial na zona II versus reparo profundo sozinho, 7 e que no grupo que teve ambos os tendões reparados acabou tendo mais reoperações por causa de aderências. 23 Outro diferencial da técnica MOP é a utilização do fio-guia que é introduzido através da incisão na polpa do dedo, evitando o alargamento das incisões na pele, além de ventilar as Polias A2 e A4, evitando danos aos tecidos moles e diminuindo o possível “efeito corda”. 23 O uso da anestesia local pela técnica WALANT, com o paciente acordado, permite observar o movimento ativo do dedo para verificar possíveis falhas no deslizamento do tendão ou formação de lacunas e, quando necessário, fazer ajustes na tensão da sutura antes do fechamento da pele.. 24

O uso de órtese ou algum tipo de imobilização é consenso em quase todos os estudos sobre técnicas de reabilitação de tenorrafia na zona II da mão. 9

Os tipos de reabilitação recomendados são geralmente baseados na natureza da lesão, no estágio de reabilitação (imediata versus regime de imobilização), na força do reparo (número de suturas no reparo), nas lesões associadas ou na capacidade de cumprir a reabilitação. 25

Uma das propostas da técnica MOP é iniciar com movimentos ativos e passivos do(s) dedo(s) lesado(s), através de orientação da equipe cirúrgica durante o intraoperatório, quando o paciente consegue visualizar e realizar movimentos de flexão e extensão do dedo reparado, que posteriormente será realizado pelo próprio paciente através de protocolo de reabilitação domiciliar ( Tabela 1 ), sem necessidade de uso de órteses, o que facilita muito a reabilitação do nosso paciente.

Neste estudo, avaliamos prospectivamente a técnica MOP, realizada por dois cirurgiões em dois centros. Há uma necessidade de avaliar sua eficácia por meio de estudos prospectivos randomizados, comparando seus resultados com outras técnicas de sutura amplamente estabelecidas na literatura, de forma multicêntrica e com um maior número de cirurgiões, a fim de verificar sua reprodutibilidade e validade externa. Nossos resultados demonstram que a técnica MOP pode ser mais uma opção dentro do arsenal terapêutico para essas lesões. A técnica de sutura MOP para reparar os tendões flexores na zona II da mão tem se mostrado segura e eficaz, com uma baixa taxa de complicações. 26
